# Supplementary material for: Densazalin, a New Cytotoxic Diazatricyclic Alkaloid from the Marine Sponge Haliclona densaspicula
Source: Molecules. 2021 May 25;26(11):3164. doi: 10.3390/molecules26113164 (PMC8198397; doi:10.3390/molecules26113164)
Supplement: Supplementary file 1 [file molecules-26-03164-s001.zip › molecules-1220899-supplementary.pdf]

Supporting Data

Densazalin, a New Cytotoxic Diazatricyclic Alkaloid from the Marine Sponge *Haliclona densaspicula*

Buyng Su Hwang[1, 2], Yong Tae Jeong[2], Sangbum Lee[1], Eun Ju Jeong[3,*] and Jung-Rae Rho[1,*]

[1] Department of Oceanography, Kunsan National University, Jeonbuk 54150, Korea; sblee08@kunsan.ac.kr
[2] Nakdonggang National Institute of Biological Resources, Gyeongbuk 37242, Korea; hwang1531@nnibr.re.kr (B.S.H.); ytjeong@nnibr.re.kr (Y.T.J)
[3] Department of Plant & Biomaterials Science, Gyeongsang National University, Jinju 52725, Korea
* Correspondence: jeong.ej@gnu.ac.kr; Tel.: +82-55-772-3224(E.J.J); jrrho@kunsan.ac.kr; Tel.: +82-63-469-4606 (J.R.R)

CONTENTS

Table S1. Relative energies and Boltzmann weights for eight conformers. --------------------3

Figure S1. Optimized low-energy conformers for **1**.---4

Figure S2. HR-ESI-TOF MS for densazalin (**1**). --5

Figure S3. ^{1}H NMR spectrum for densazalin (**1**). --6

Figure S4. ^{13}C NMR spectrum for densazalin (**1**). ---7

Figure S5. COSY NMR spectrum for densazalin (**1**). --8

Figure S6. TOCSY NMR spectrum for densazalin (**1**).---9

Figure S7. HSQC NMR spectrum for densazalin (**1**). --10

Figure S8. HMBC NMR spectrum for densazalin (**1**).--11

Figure S9. NOESY NMR spectrum for densazalin (**1**). --12

Calculation of ECD spectrum of 1.

The configurational structure of densazalin (**1**) determined by NMR spectroscopy was used as an input for a conformational searching of **1**. The conformational search was performed by spartan 18 software, which calculates with molecular mechanics. Eight conformers with low energies were selected within a 11 kJ/mol threshold. Each conformer was optimized by the DFT method at the B3LYP/6-31G(d) level using Gaussian 16 program. Following this procedure, the ECD spectrum of each conformer was calculated by the TD-DFT method at the B3LYP/6-31G(d,p) level with the PCM model in methanol solvent. The weights of the conformers by Boltzmann distribution were obtained from the calculation of single-point energy of eight conformers at the B3LYP/6-311G+(2d,p) level.

Table S1. Relative energies and Boltzmann weights for eight conformers.

conformers	Electronic energy (au)	Relative energy (KJ/mol)	Boltzmann weights (%)
1	-1357.326787	0.00	46.3
2	-1357.326328	1.20	28.4
3	-1357.325550	3.25	12.4
5	-1357.324527	5.93	4.2
6	-1357.324257	6.64	3.1
4	-1357.324228	6.72	3.1
7	-1357.323725	8.04	1.8
8	-1357.322750	10.60	0.6

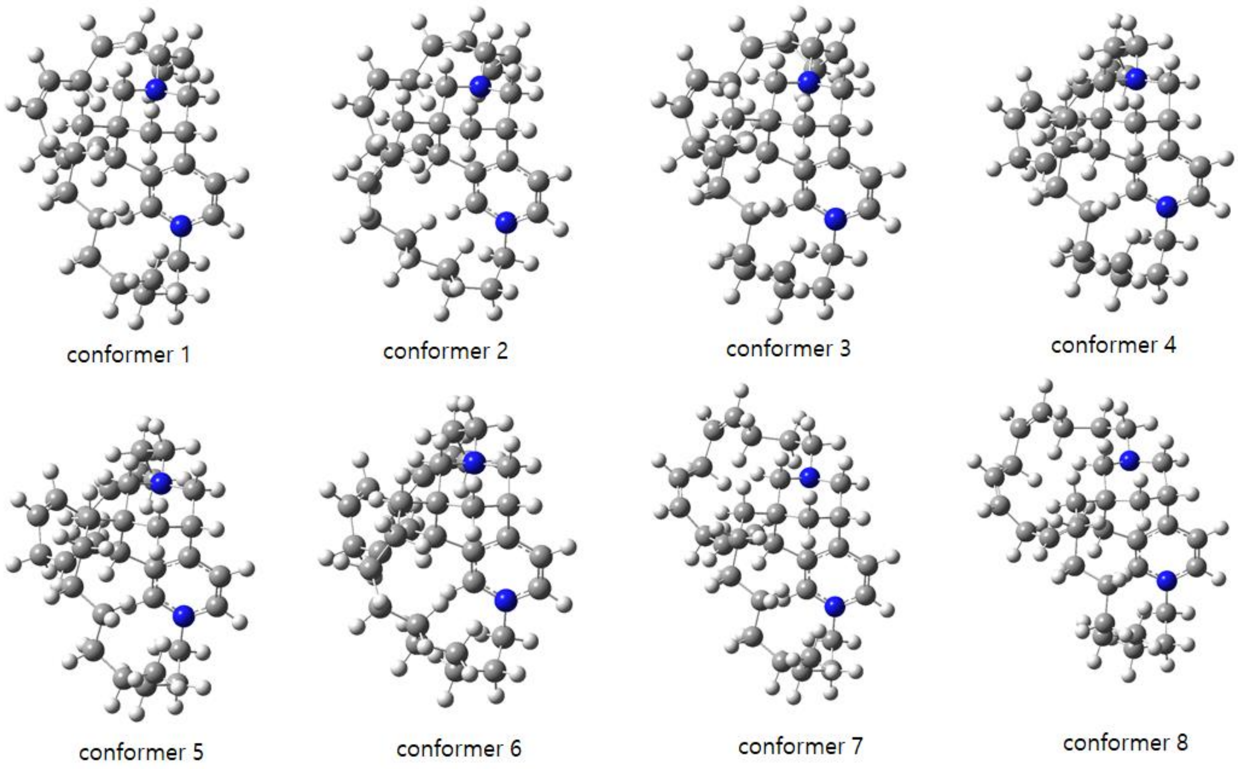

Figure S1. Optimized low-energy conformers for **1**.

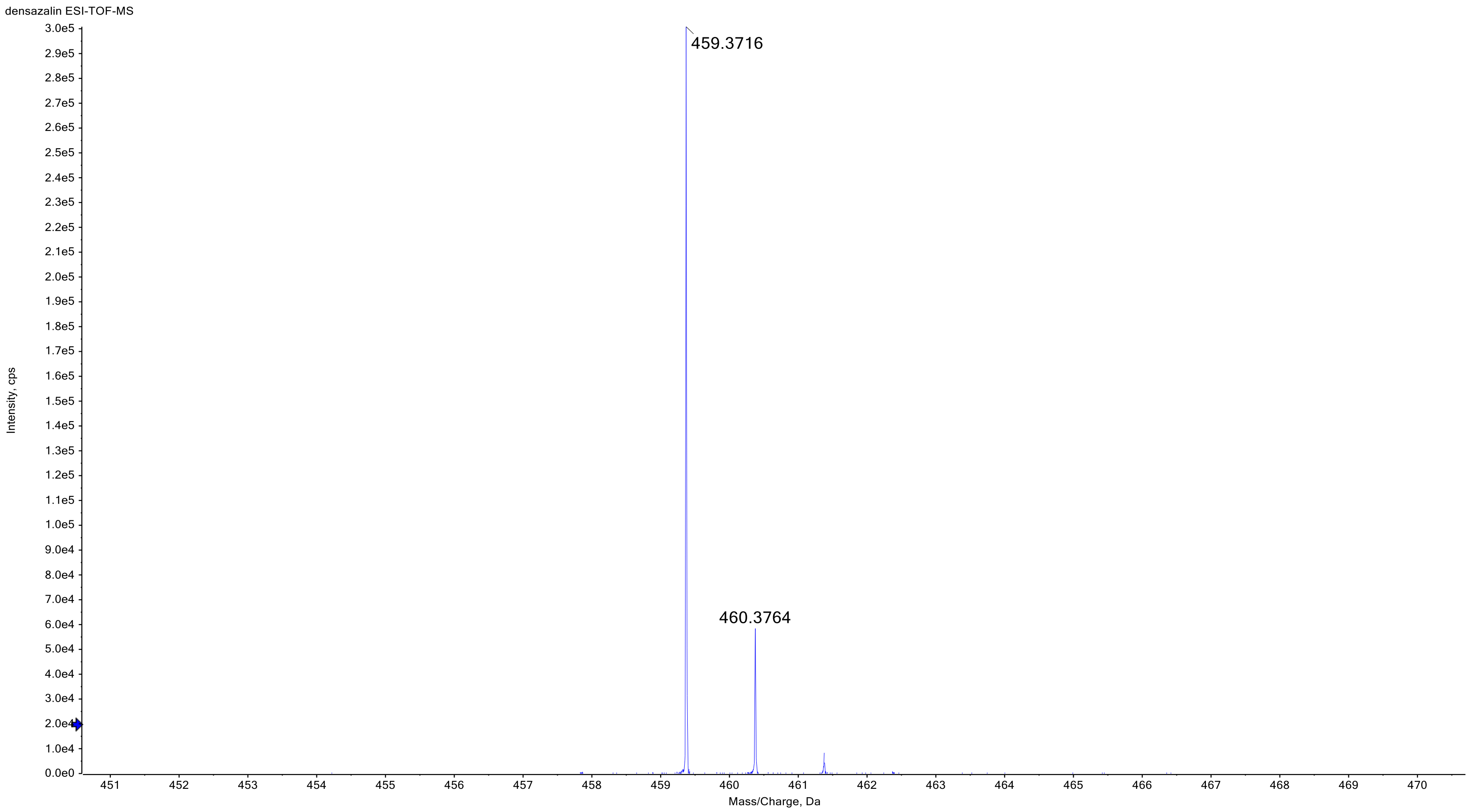

Figure S2. HR-ESI-TOF MS for densazalin (**1**).

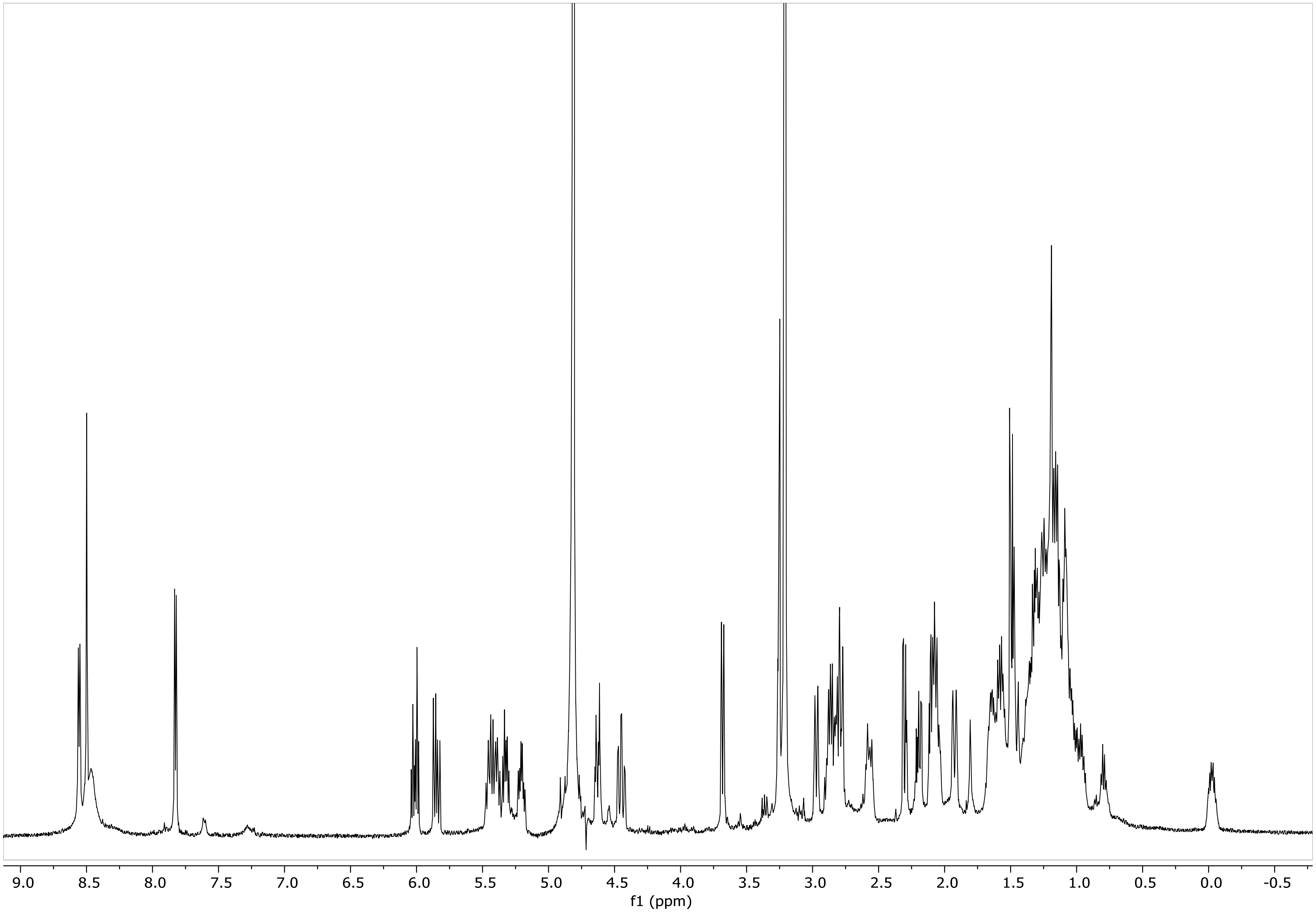

Figure S3. ^{1}H NMR spectrum for densazalin (**1**).

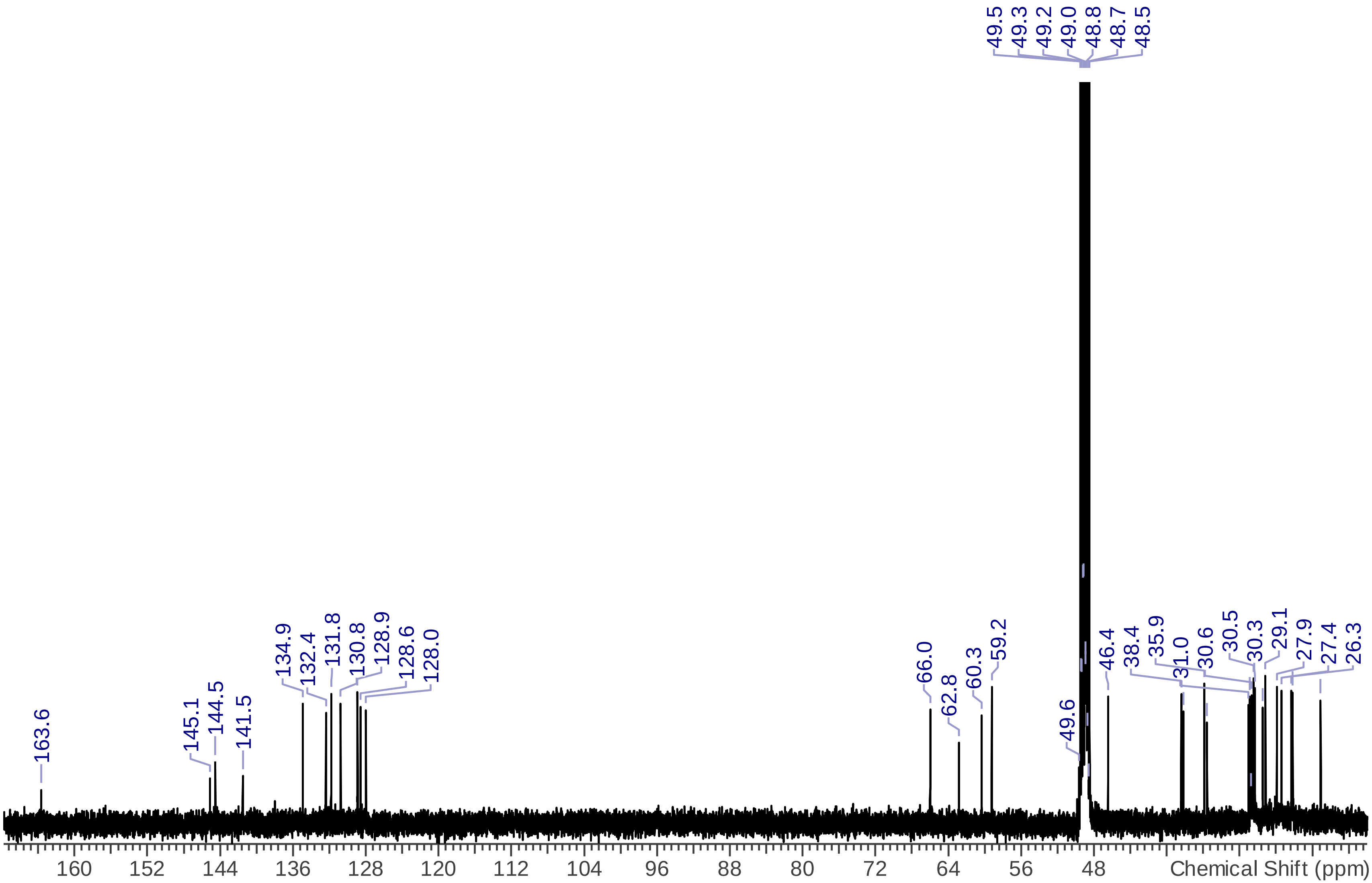

Figure S4. ^{13}C NMR spectrum for densazalin (**1**).

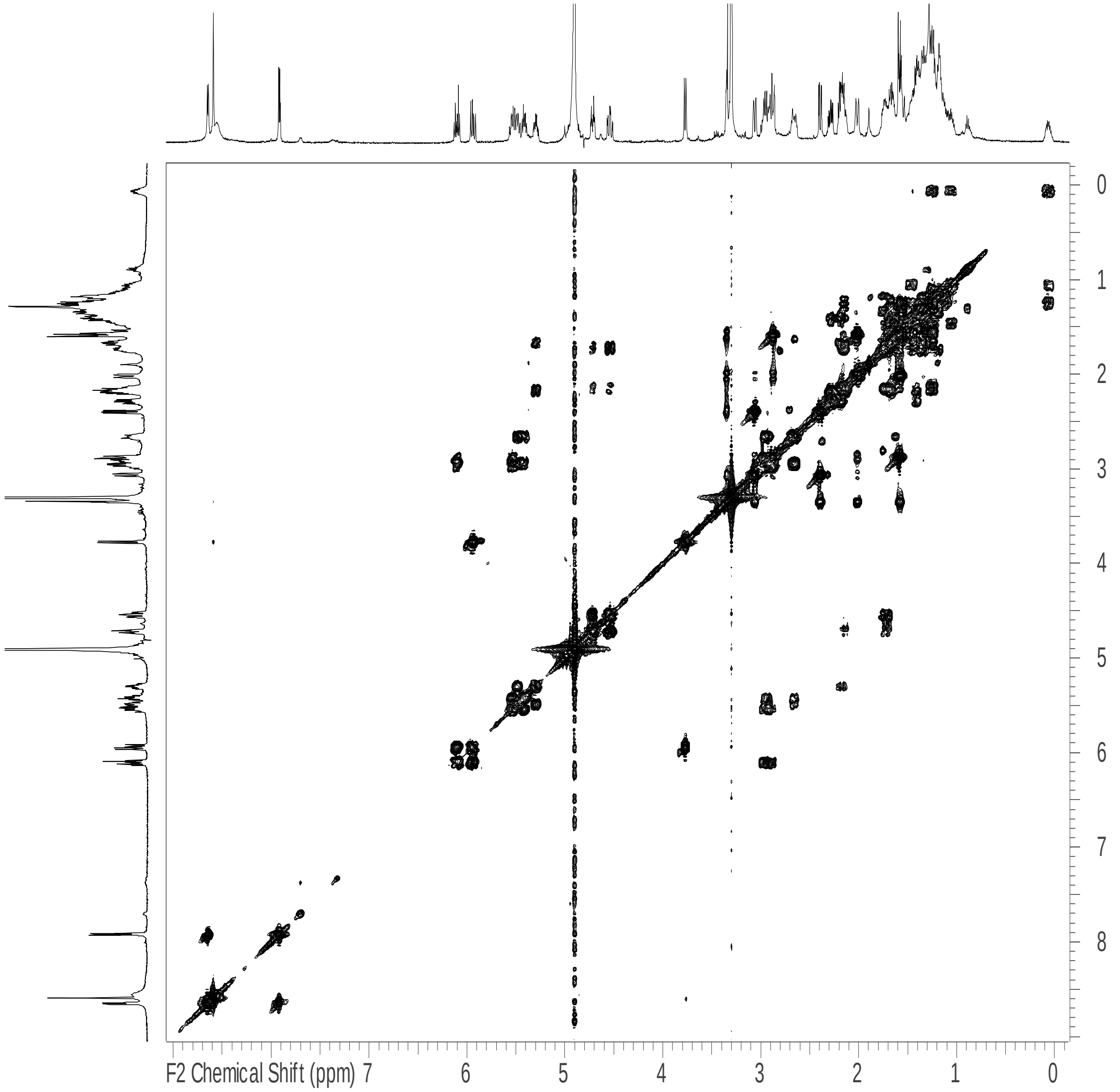

Figure S5. COSY NMR spectrum for densazalin (**1**).

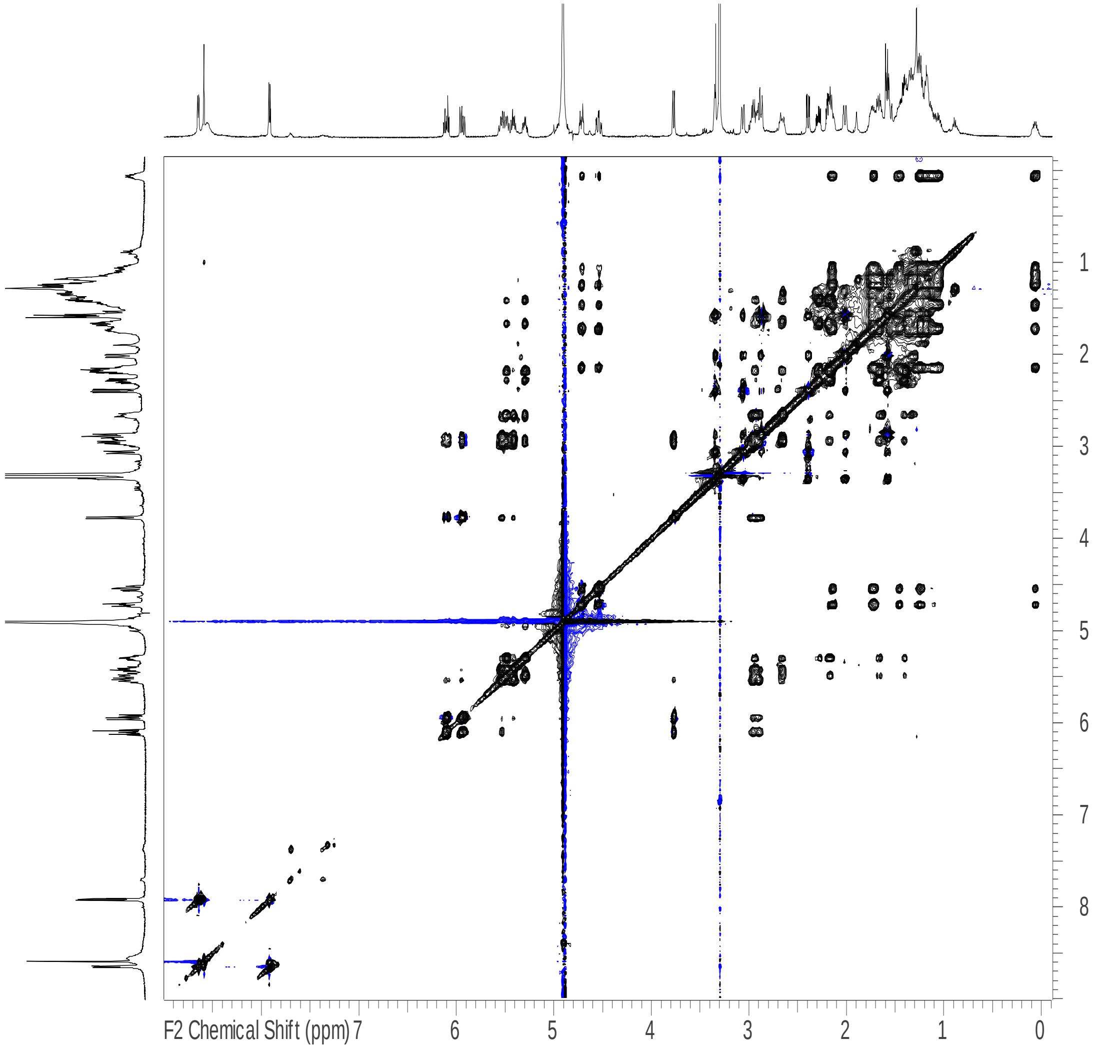

Figure S6. TOCSY NMR spectrum for densazalin (**1**).

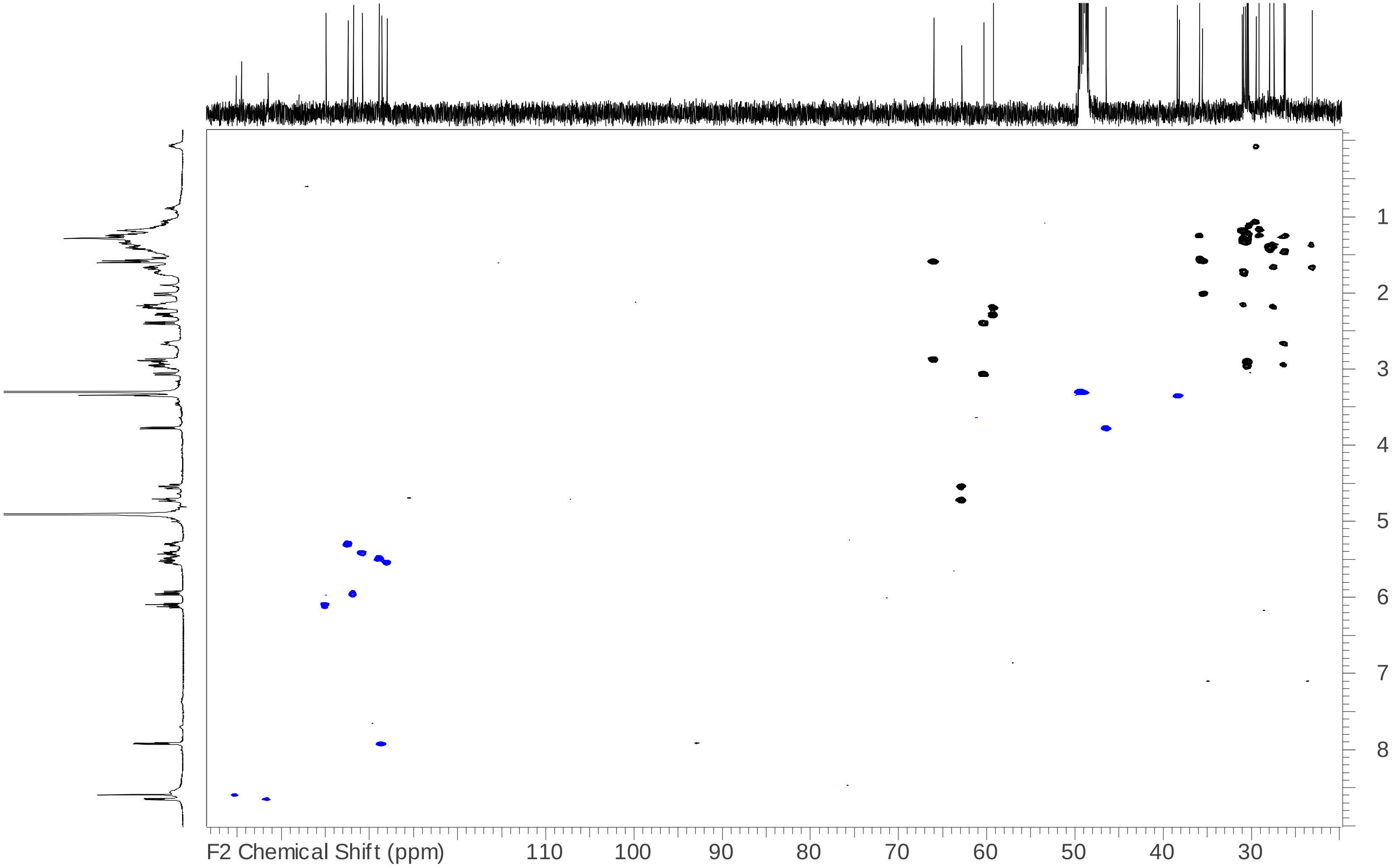

Figure S7. HSQC NMR spectrum for densazalin (**1**).

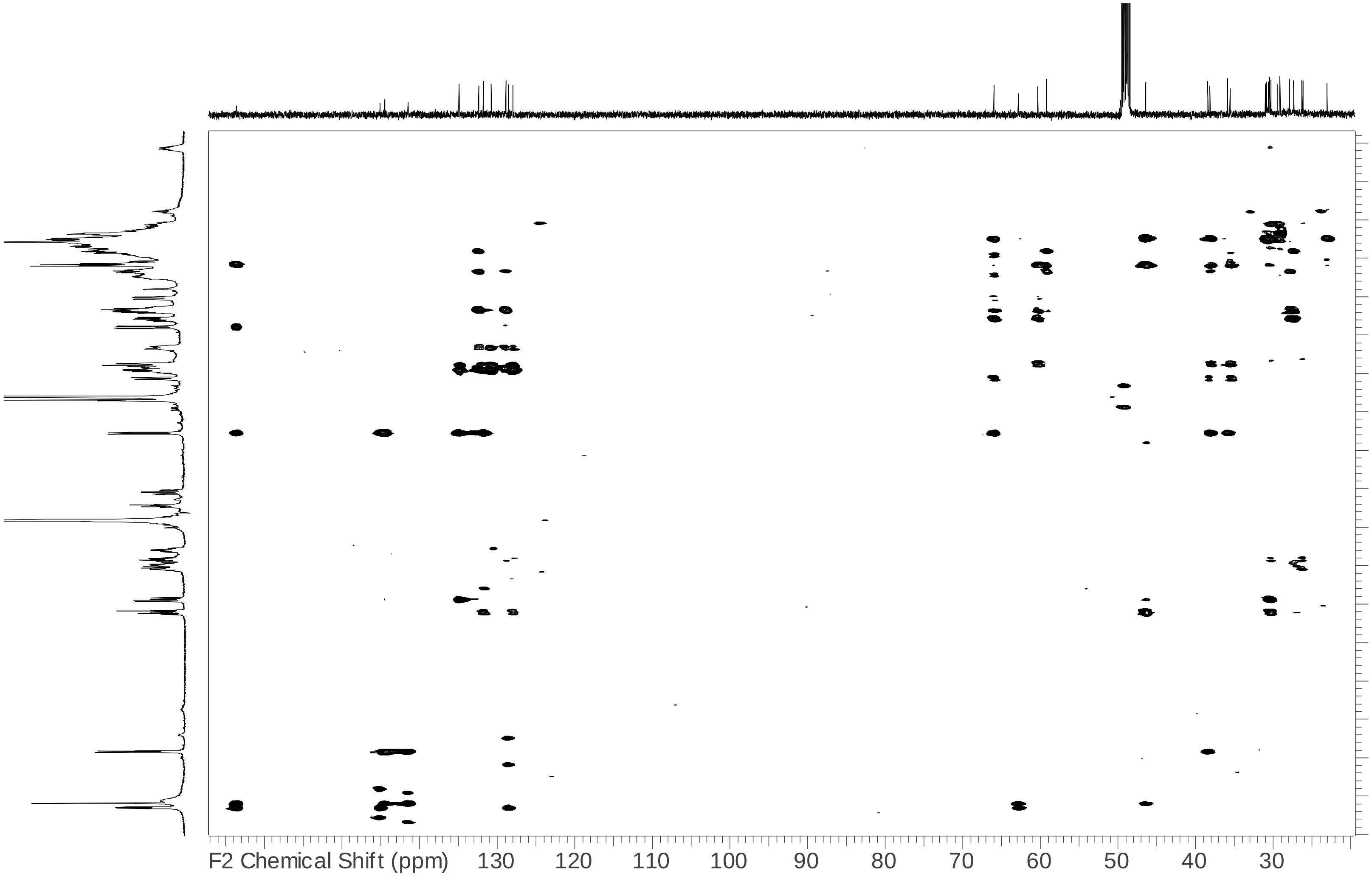

Figure S8. HMBC NMR spectrum for densazalin (**1**).

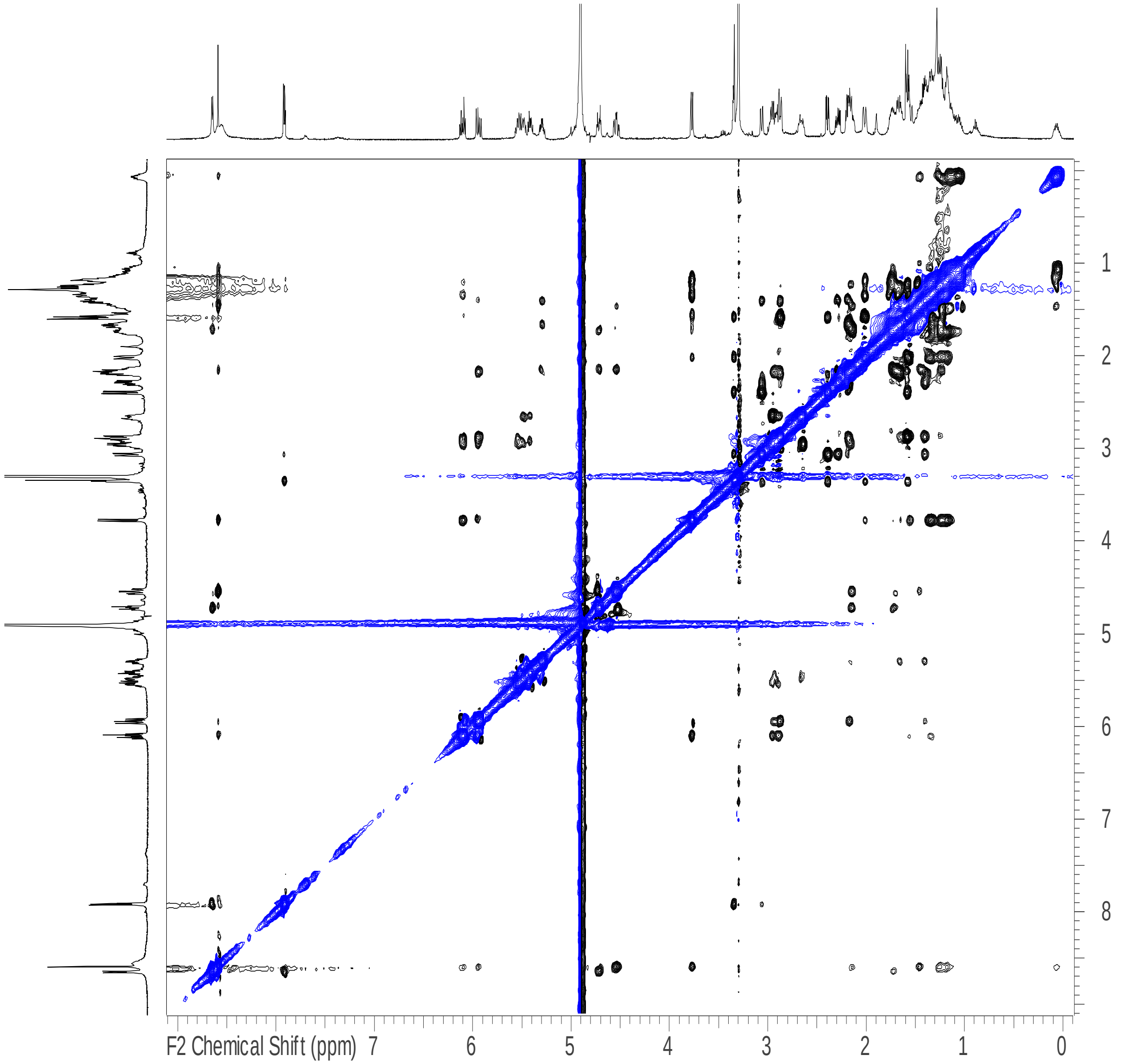

Figure S9. NOESY NMR spectrum for densazalin (**1**).
